# The biopsychosocial effects of transtibial amputation: A South African perspective

**DOI:** 10.4102/ajod.v14i0.1404

**Published:** 2025-04-30

**Authors:** Riyona Chetty, Raisuyah Bhagwan, Nalini Govender

**Affiliations:** 1 Department of Medical Orthotics and Prosthetics, Faculty of Health Sciences, Durban University of Technology, Durban, South Africa; 2 Department of Community Health Studies, Faculty of Health Sciences, Durban University of Technology, Durban, South Africa; 3 Department of Basic Medical Sciences, Faculty of Health Sciences, Durban University of Technology, Durban, South Africa

**Keywords:** amputation, quality of life, biopsychosocial, phantom limb pain, support, feelings, body image

## Abstract

**Background:**

A myriad of physical, psychosocial and environmental sequelae are associated with limb loss. However, there is a paucity of empirical South African data, which focusses on these sequelae, how they interface with the amputee’s quality of life as well as the challenges they experience following amputation.

**Objectives:**

This study sought to explore the biopsychosocial effects of amputation and how it affected the quality of life of transtibial amputees.

**Method:**

A qualitative approach guided this study. Data were collected using one-on-one interviews with 14 unilateral transtibial amputees. Data were analysed thematically.

**Results:**

Five broad themes emerged from the inquiry, which captured amputees’ experiences of phantom limb pain, body image disturbances and their challenges related to adapting to daily activities. Participants also expressed the salience of familial support as well as the importance of psychological interventions to cope.

**Conclusion:**

The findings suggested that support networks and professional psychological intervention are imperative in facilitating successful adjustment to the amputation experience. Raising awareness of limb loss, in both rural and urban settings, may help reduce the stigma attached to it.

**Contribution:**

Quality of life comprises several domains, namely physical, psychological, environmental and social. However, limited local and international data exists regarding the environmental and social effects. This study brought to the fore the positive and negative effects of amputation in each domain, as well as various strategies, which facilitate successful adjustment to amputation.

## Introduction

The ability to stand, ambulate and run upright on two lower extremities is one of humanity’s defining physical characteristics, which enables humans to navigate their environment (Farris et al. 2019:1645; Handford 2018:1). Hence, the removal of the lower limb because of a traumatic event or debilitating disease, significantly compromises the individual’s mobility and functional independence (Boyd 2020:1; Day, Wadey & Strike 2019:2435; Handford 2018:1; McDonald 2017:6).

Diabetic neuropathy, poliomyelitis, osteomyelitis, dysvascularity, malignancy, motor vehicle accidents, work-related traumas and infections are common reasons for lower extremity amputations (Grzebień et al. 2017:57; Khan et al. 2020:1437; Knežević et al. 2015:103; Matos, Naves & Araujo 2020:2). Lower limb amputations are prevalent surgical procedures, accounting for an estimated 31.7% of traumatic amputations worldwide (Hawkins et al. 2014:763; McDonald et al. 2020:4). Data from South Africa indicate that transtibial amputations are the most prevalent type, as a result of complications arising from poorly controlled diabetes mellitus, followed by atherosclerosis and traumatic events (Khan et al. 2020:1437; Manickum, Ramklass & Madiba 2019:44; Olotu & Anderson 2019:10).

Irrespective of the cause, amputation of the lower extremity triggers salient psychological, environmental, vocational, physical and social sequelae, which may negatively affect the individual’s quality of life (Gonçalves Jnr., Knabben & Da Luz 2017:98; Knežević et al. 2015:103; McDonald 2017:6). Despite the range of challenges associated with adjusting to an amputation, the potential for psychosocial growth is reported (Flinn 2016:11; Godlwana & Stewart 2013:48; Jefferies 2015:29). Hence, a well-designed, patient-specific rehabilitation programme combined with the knowledge, skills and expertise of the multidisciplinary team can help mitigate these challenges (Magnusson 2014:31).

According to the World Health Organization (WHO), quality of life can be defined as one’s subjective view of one’s own life in the context of the value systems and culture in which a person lives (Knežević et al. 2015:103; The WHOQOL Group 1998:1572). The degree to which one is comfortable, healthy and able to participate in or enjoy life events also contributes to one’s quality of life. This ambiguous phrase is multipronged and encompasses the physical, psychological, social and environmental domains (The WHOQOL Group 1998:1572). Each of these domains consists of facets that overlap, each contributing to one’s comprehensive quality of life.

Multiple challenges are associated with amputations. Data emanating from studies conducted in England (Washington & Williams 2016:44), Serbia (Knežević et al. 2015:106) and India (Srivastava & Chaudhury 2014:4) suggest that amputees experienced feelings of depression and anxiety as well as having to embrace an altered body image. Social discomfort was identified as another key experience in Manchester (Washington & Williams 2016:49) and Edinburgh (Uytman 2014:15). Noteworthy, phantom limb pain was reported as a common encounter by amputees in London (Trevelyan, Turner & Robinson 2016:70), India (Bhutani et al. 2016:10) and Vojvodina (Knežević et al. 2015:106) following an amputation.

Environmental barriers, financial burdens and poor access to healthcare services also contributed to altering the amputee’s quality of life, particularly in Iran (Abdi et al. 2015:1481) and Vojvodina (Knežević et al. 2015:104). Activity restrictions, loss of independence and unemployment were additional challenges experienced by the amputees in India (Bhutani et al. 2016:10) and Ireland (Coffey et al. 2009:1066). Similar South African findings were reported in the Eastern Cape (Manig 201879), KwaZulu-Natal (KZN) (Ramkisson, Pillay & Sartorius 2016:4), Western Cape (Ennion & Rhoda 2016:565; Yu & Ennion 2019:3;) and Gauteng (Godlwana 2015:133; Godlwana & Stewart 2013:51) provinces.

In South Africa, amputations are known to be accompanied by the loss of independence (Boyd 2020:1), unemployment, exorbitant medical bills and intolerable pain (Abdi et al. 2015:1481). Despite the assurance of having the right to access healthcare services being stipulated in Section 27 of The Constitution of the Republic of South Africa (South Africa, Department of Justice 1996:11), there is still limited access to healthcare services, inconsistencies in resource provision and consequent lack of prosthetic services to amputees (Naidoo & Ennion 2019:102). This has subsequently led to the onset of depressive symptomatology in several amputees who consequently do not receive psychological support (Amputee Coalition 2020:para 1 line 5). Environmental barriers comprise lengthy queues and travel distances especially from rural areas, exorbitant healthcare service and travel costs (Naidoo & Ennion 2019:98). Often, the uninsured individuals and rural citizens are unable to afford the healthcare they require or are entitled to (Naidoo & Ennion 2019:96). This limited access to healthcare among amputees results in poor work performance and a reduced lifespan.

There is a growing consensus that the lack of data regarding the quality of life following an amputation is a gap in South African literature (Godlwana & Stewart 2013:49). This study therefore sought to understand the biopsychosocial effects experienced by transtibial amputees and how it affects their quality of life. The findings are directed towards potentially offering motivation to current and future amputees.

### Significance of the study

The physical and psychological impediments of a lower limb amputation have been widely researched (Amoah et al. 2018:2; Godlwana & Stewart 2013:50; Paul 2018:2; Roșca et al. 2021:2; Varga & Gallagher 2020:187; Zhu et al. 2020:3). However, there is a paucity of local and international data regarding the environmental and social effects. This study seeks to close the gaps in literature by exploring how amputation is affected by each domain.

Gaining an understanding of the constituents attached to limb loss from the vantage point of the amputee will assist prosthetists in the dissemination of relevant information pre- and post-operatively regarding the prosthesis, the patient’s expectations and goals, as well as any other aspect of vital concern to the patient. There is potential that prosthetists will be encouraged to enhance the current education regarding the adjustment process that the patients experience as well as provide resources to current and future amputee patients. Additionally, prosthetists can consider support regarding coping strategies, which will cater for a more holistic management structure. Furthermore, this comprehensive understanding will assist the prosthetist to acknowledge the importance of referrals, to efficaciously provide quality patient care to individuals with limb loss and to improve accessibility of their services in rural areas (Ennion & Rhoda 2016:565; Manickum et al. 2019:44). Each of the aforementioned factors will contribute to the quality of life of amputees and assist prosthetists in improving their clinical expertise, thereby allowing prosthetists to establish a good rapport with patients. Moreover, the findings have potential to encourage prosthetists to enhance the patient experiences regarding frequent prosthesis use, satisfaction and quality of life.

### The conceptual framework

**FIGURE 1:**
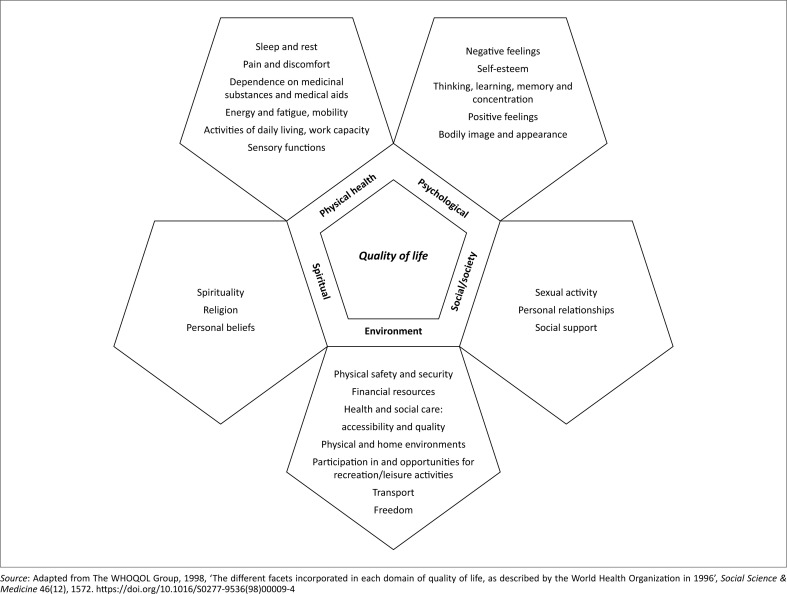
The different facets incorporated in each domain of quality of life, as described by the WHO (1996:5).

The quality of life of individuals with an amputation was positioned at the core of this study. Therefore, the facets of each domain of quality of life played a crucial role in determining the most accurate conceptual model to guide this study. The facets incorporated in each domain of quality of life are illustrated in Figure 1.

### The International Classification of Functioning, Disability and Health

This research study was guided by The International Classification of Functioning, Disability and Health (ICF), which is a universal language and conceptual foundation for the description and measurement of disability, developed by the WHO. The ICF is a multidimensional model, which can be used as a powerful tool for research in the health sector, among other various purposes and uses in several different sectors (WHO 2013:1).

The ICF is a ‘bio-psycho-social synthesis’ as it was developed by the integration of the two main disability models: the medical model and social model (WHO 2013:5). The framework can be used to arrange and document material on disability and functioning. The ICF definitions and categories are worded objectively to allow for both positive and negative aspects of functioning to be recorded (WHO 2013:5).

**FIGURE 2:**
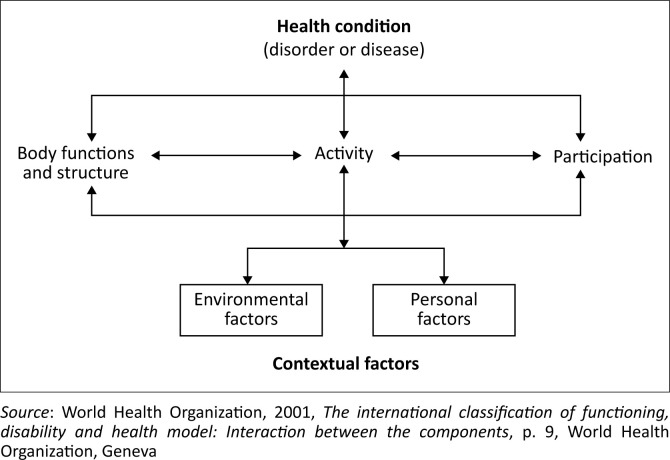
The international classification of functioning, disability and health model: Interaction between the components.

The ICF model (Figure 2) is structured in two parts, which are further sectionalised (WHO 2013:7):

Part 1- Functioning and Disability:

- Body functions and body structures
- Activities and participation

Part 2- Contextual Factors:

- Environmental factors
- Personal factors

It is critical to note that the data respective to the above-mentioned entities must be collected independently and then explored for relationships between them (WHO 2013:7).

The ICF model, as illustrated in Figure 2, was adopted as it provides a systematic, effective basis for explaining, understanding and examining health and health-related states, effects and causal factors (WHO 2013:6). The ICF was only employed as a conceptual model for this study; the domains and facets were used to describe functioning without the utilisation of the particular ICF codes or categories. The domains can be assumed as important sets of body functions, tasks, actions or areas of life, which capture a specific phenomenon or the experiences of an individual (WHO 2013:23).

## Research methods and design

It was imperative to the researcher that the anonymity and confidentiality of the participants are respected and maintained. Specific measures were implemented to protect the identity and data pertaining to the participants as well as the data collected. The researcher created and allocated a random pseudonym for each participant, which was a combination of a numeric value and two letters of the alphabet. Hard copy data were locked in the researcher’s home cabinet for the duration of the study. All the hard copy data will be shredded by the researcher after 5 years. Electronic data were stored on the researcher’s laptop and encrypted. Further, the electronic data will be destroyed by the researcher after 5 years. This is in keeping with DUT research protocols.

### Study design and setting

When the research problem is clear, unambiguous and known or when factual data or probability information is required in psychological research, a quantitative research design is most appropriate (Hammarberg, Kirkman & De Lacey 2016:498). The current study was devoted to acquiring insight on how individuals with limb loss make sense of the amputation experience; therefore a qualitative research approach was adopted. Said approach is participant orientated as it is used to understand, explore and describe feelings and experiences (Kothari 2004:3). Qualitative research involves an interpretive naturalistic approach to its subjects (Denzin & Lincoln 1994:2). An advantageous element of a qualitative design is that a ‘bonding relationship’ is allowed to form between the researcher and the participants (Alase 2017:9). This reiterates the study’s primary interest of obtaining a detailed understanding and interpretation of the human experience related to the psychosocial, environmental and physical challenges faced by amputees. These raw emotions can be richly received through a qualitative inquiry that allows the lived experiences of participants to be reflected in a descriptive style (Alase 2017:10). The study was exploratory in nature, as it was undertaken to gain a deepened understanding of the essences of living with amputation. Exploratory research was appropriate as it seeks to answer questions such as why, what and how (Stebbins 2001:2).

This study was conducted at one of the Department of Health’s facilities in the greater eThekwini Metropolitan Municipality, KwaZulu-Natal province. The medical facility in question has several departments that provide quality care and service to patients, namely dietetics, psychology, physiotherapy, mental health (psychiatry clinic), prosthetics and orthotics, family medicine and social work. Also located at the facility is a sectioned-off institute established by the DUT offering a Work Integrated Learning programme for medical orthotic and prosthetic students.

### Study population and sampling

The sample was selected based on a specialised set of characteristics that they possessed, which in this case was a group of unilateral transtibial amputees. In the context of this study, unilateral indicates that a part of only one limb is amputated, and transtibial describes the amputation level that is through the tibia. Participants were drawn from a list of transtibial amputees scheduled for appointments at the respective medical facility between August and September 2021.

Aligned with qualitative research approaches, a smaller sample was selected to attain information richness with regard to exploring the biopsychosocial effects of an amputation experienced by individuals with limb loss. The researcher used purposive sampling, which is a non-probability sampling strategy. The sample consisted of 14 transtibial amputees of whom 10 were males and 4 females. Predominantly, there were eight Black participants, followed by five Indian participants and one White participant. Recruitment was completed after data saturation was reached.

Data saturation is reached when there is sufficient data collected to replicate the study (Fusch & Ness 2015:1408), and the data no longer discloses new properties of the researcher’s core theoretical categories. If at this point not all the participants have been interviewed, they will still be interviewed for purposes of trustworthiness (Dworkin 2012:1319). Although an initial sample of 10 participants was identified, data saturation was not achieved. Four additional participants were interviewed and thereafter when no further new themes emerged, interviews stopped. Saturation was achieved at this point.

### Recruitment and data collection

The flyer, printed in English and in isiZulu, was displayed at the orthopaedic department and provided pertinent details of the study. If the amputee wished to participate in the study, they contacted the researcher whose number was displayed on the flyer or informed their prosthetist. Once contact was established, an interview date was set. Because of coronavirus disease 2019 (COVID-19), several patients chose not to attend their follow-up appointments. Hence, the secretary in the Orthopaedics Department at the medical facility assisted in the recruitment of patients on the amputee database using the inclusion and exclusion criteria delineated for the purposive sampling of participants. The amputee participants were recruited based on the following inclusion criteria: unilateral transtibial amputee (long, standard or short), ages between 25 and 75 years, amputation surgery was performed before January 2018, regular use of the prosthesis over a period of 1 year and no symptoms of post-traumatic stress disorder.

Prospective participants were identified by the secretary via review of the amputation levels recorded in the medical profile, as well as by the prosthetist during the follow-up appointment. The secretary subsequently informed those who met the inclusion criteria of the study. The researcher telephonically contacted all consenting participants and arranged for a suitable interview date. All the participants were informed that the interview would take place either at their home or the medical facility. In addition, they were given the option of virtual interviews either through telephonic contact, a Microsoft Teams call or a WhatsApp videocall.

The researcher was not involved in the recruitment process, thus, mitigating self-selection bias. There were no participants that withdrew from the study or chose not to participate after reading the information letter related to the study objectives. The researcher was unaware if patients denied potential participation once asked by the prosthetist, as only those willing to participate were in contact with the researcher.

Semi-structured interviews were chosen as the appropriate data collection technique for the study because the intent was to understand the effects of amputation on the participants. The flexibility of this approach allows the researcher to discover or elaborate on material that may not have previously been thought of by the researcher (Neuman 1997:33).

The researcher conducted in-depth interviews over a period of approximately 2 months. All 14 participants were interviewed face to face; 10 were interviewed at the medical facility and 4 at the participants’ homes. The interviews were audio recorded and then manually transcribed by the researcher.

### Data collection instruments

The following research instruments were used during data collection, namely the researcher, an interview guide, an audio recorder and a reflex diary.

The qualitative researcher methodically observes individuals and experiences with the purpose of discerning and learning about behaviours and communications in natural settings. Such observation exemplifies the idea of the researcher as the key instrument of the qualitative inquiry. It entails going into specific settings and detailing and analysing what has been observed. This valuable method has been informative and insightful in health care settings (Mays & Pope 1995:182). As the researcher was the principal instrument, sufficient training, experience and careful preparation were undertaken prior to data collection to ensure researcher credibility (Patton 1999:1198).

An interview guide was used to collect data from the sample. Formulated before the interviews, the guide comprised of a list of high-level topics and questions with which the researcher could direct the conversation (Knight 2013:1). These topics centred on the research questions. The guide assisted the researcher to focus, organise their line of thinking and in what sequence to pose the questions (Kennedy 2006:1). The interview guide used for the sample included an English and isiZulu translation version. The guide included questions regarding pre- and post-operative activities, challenges faced while using the prosthesis, the patient-clinician relationship and the quality of the prosthesis.

A reflexive diary is a valuable research tool that can be defined as a written or verbal personal record transcribed by the researcher. During the interviews, observations of participant emotions and reactions were noted in the reflexive diary that assisted the researcher in contextualising the participants’ experiences and enhanced the researcher’s understanding of the data.

The researcher was aware of potential bias considering her professional background as a prosthetist. Being acutely aware of this, the researcher did not allow this to influence data collection and analysis. As advised by Berger (2015:231), the researcher continually self-reflected and constantly updated her position relative to the study and discussed this with the co-investigators and how it could influence the study findings. Excerpts evidencing this are as follows:

‘My prosthetic background may drive me towards interpreting findings through this lens. I must remain cautious and take into consideration alternative explanations that may not align with my prior professional experiences.

During coding of themes, I must continually seek feedback from my co-coders to ensure accurate interpretation of the data without influence of my personal biases.’ (Chetty, Female, 25)

Audio recordings are a simple and effective technique that provides the researcher with a record of the interview, which is a valuable reference when transcribing the interviews. The researcher is liberated from the distracting task of notetaking during the interview and can focus on interacting with the participant. An audio recording technique ensures a verbatim and accurate transcription of the interview (Whiting 2008:37). Considering these benefits, a digital recorder was used during the one-on-one interviews. The researcher obtained written consent from all the participants to audio record the interviews.

### Data analysis

Thematic analysis was used to guide the analysis. It is the most common method for qualitative studies and focusses on identifying the recurring issues and main themes that emerge from the data obtained (Braun & Clarke 2006:78). The main themes should summarise all the views that the researcher has collected. Thematic analysis offers a particularly flexible approach, which can be modified according to the needs of a research study, thereby providing a thorough and rich yet intricate report of data (Nowell et al. 2017:2). This method was chosen because it provides a truly comprehensive analysis and ensured that the analysis did not only concentrate on the atypical extracts of the data (eds. Bricki & Green 2007:25).

The following steps were completed to thematically analyse the data:

#### Step one: Familiarisation with the data

Preliminary observations were made. This was particularly useful with the first few transcripts where the researcher was still trying to get a feel for the data. The transcripts were read and re-read. The researcher made notes and jotted down any impressions.

#### Step two: Generating initial codes

Next, the data are organised in a meaningful and systematic way. The researcher utilised open coding, which implied that there were no pre-set codes. The codes were developed and modified as the researcher worked through the coding process. Each segment of the transcript that was relevant or significant to or addressed the research question was coded.

#### Step three: Searching for themes

A theme is a pattern that captures something significant or interesting about the data and/or the research question. The researcher examined the codes to identify common and recurring themes that were generated in the study. The themes were predominantly descriptive, which meant that the described patterns in the data were related to the research question. The codes were either associated with one particular theme or more than one depending on the data collected.

#### Step four: Reviewing the themes

During this stage, the preliminary themes were reviewed, modified and developed. All the data relevant to each theme were gathered using Microsoft Excel. The researcher then considered whether the data associated with each theme supported it and then deliberated how the themes worked within a single interview and across all the interviews.

#### Step five: Defining the themes

In the final refinement of the themes, the aim was to identify what each theme was about. The researcher constantly and intensely scrutinised the themes and sub-themes. This deep level of total engrossment allowed the researcher to gain insight into the personal experiences of the participants by becoming thoroughly immersed in the data collected. Further perusal of the themes and sub-themes generated, allowed the researcher to check for correlation within them.

#### Step six: The write-up

Finally, the researcher wrote up the analysis of the findings made (Braun & Clarke 2006:87).

### Strategies used to achieve trustworthiness

The four criteria of credibility, confirmability, dependability and transferability were used to ensure the trustworthiness of the study (Guba 1981:80).

Credibility was achieved through triangulation. The researcher also ensured that all the participants’ experiences, including divergent responses, were respected and reflected in the presentation and analysis. According to Shenton (2004:68), checking of data may take place at the end of the data collection or even at the end of each interview. Member checking occurred after the interview session where the participants and researcher listened to the audio-recorded dialogue of the interview to allow the researcher to check that the participant’s responses were clearly understood. Following the interview session, the participants and researcher listened to the audio recorded dialogue of the interview to allow the researcher to check that the participant’s responses were clearly understood. Further, participants were able to consider whether their statements have been clearly and correctly communicated. This ensured that accurate transcriptions occurred. For the interviews with isiZulu-speaking participants, the translator was present during member checking. A prosthetist colleague, not involved in the study, checked the researcher’s interpretations of the data to further ensure the credibility of the findings.

Confirmability was ensured through consistent maintenance and perusal of notes made in the reflexive diary, as well as established via an audit trail. This showed that the participants’ responses were accurately portrayed in the findings and highlighted each step of data analysis that was performed in order for a rationale to be provided for the decisions made (Houghton et al. 2013:14).

Dependability can be established with inquiry audit, which entails an outside individual (auditor) reviewing one’s study to verify that results were consistent and authentic, and one’s study can be repeated (Houghton et al. 2013:14). After data collection, the findings were presented to a prosthetist colleague to validate them.

The primary investigator assigned codes to the data using the study’s conceptual framework and intercoder reliability (ICR) as an essential guide and criterion to promote reflexivity within the research team, improving communicability and transparency of the coding process (O’Connor & Joffe 2020:11). Themes were then developed from the codes. At each stage, the co-coders consistently cross-checked and validated the coding decisions that ensured coder reliability and increased the robustness of the coding.

Detailed and appropriate information pertaining to the study’s context, research methods and primary data was provided for the reader to evaluate the transferability of the findings. Verbatim quotes from the participants were used to create a rich understanding of the context for the reader. To enhance transferability, a detailed presentation of the findings was produced.

### Ethical considerations

Full ethical approval was obtained from the Institutional Research Ethics Committee (IREC 119/20) at Durban University of Technology (DUT) on 22 December 2022. All procedures performed in studies involving human participants were in accordance with the ethical standards of the institutional and/or national research committee and in accordance with the 1964 Helsinki Declaration and its later amendments or comparable ethical standards. Participants were made aware of two important facts, namely that their participation in the study was on a voluntary basis and that they could withdraw from the study at any point in time. Written informed consent was obtained from each participant to ensure that they were fully aware of the details of the study and what was required in terms of their participation. No coercion was used during the recruitment. In addition, no psychological or emotional distress was inflicted on any participants during the interviews. The researcher was sensitive to the power they held during the research process and was hence respectful towards the participants.

## Results and discussion

Five broad themes emerged from the data that captured the nuances of the adjustment to amputation and how the quality of life of individuals enduring limb loss was affected. Participant responses illuminating the post-amputation effects are described under the following themes: phantom limb pain, psychological issues, adaptation to daily activities, familial obligations and responsibilities and stereotyping in society.

### Theme 1: Phantom limb pain

The first theme that was derived from the data focussed on phantom limb pain:

‘I have phantom limb pain all the time. It is so painful and I even cry. It feels like someone is cutting me with a knife. The medication is addictive so it can’t help me.’ (AP12, Male, 25)

The amputees had different experiences with phantom limb pain depending on their level of pain tolerance and method of pain relief. Participant AP12 felt the pain to be unbearable especially after learning that one of the side effects of his medication was addiction. Hence, the pain may have been intensified by the participant’s frustration of not having an effective pain relief method. A study done in North Carolina found that amputees felt the side effects of pain relief medication were worse than the phantom limb pain itself and therefore, no longer used the medication (Paul 2018:11).

Another participant found beneficial home remedies to seek relief:

‘I still get phantom limb pain but not all the time. I splash cold water on the leg and it helps with the pain.’ (AP7, Female, 74)

Participant AP7 indicated that colder weather temperatures would trigger the phenomenon. This sensation was corroborated by Stockburger, Sadhir and Omar (2016:161) who pointed out that colder temperatures reduce peripheral blood circulation that could precipitate pain in the residual limb.

Participant AP14 stated that his phantom limb pain was exacerbated by delayed wound healing as a hindrance of poorly controlled diabetes:

‘I did have phantom limb pain and it felt like a ghost. The pain lasted for three years. As a diabetic, the wounds took long to heal so the pain was quite bad.’ (AP14, Male, 71)

Spampinato et al. (2020:3) and Patel et al. (2019:11) highlighted that poor wound healing was a common consequence endured by amputees with diabetes because of hyperglycaemia, reduced vascular circulation, neuropathy and impaired immune response. Andjić et al. (2021:2) further stressed the importance of proper wound management in amputees with diabetes, saying it would drastically enhance their quality of life and decrease morbidity and mortality.

Current literature reflects that phantom limb pain is a common occurrence affecting a multitude of individuals post-amputation (Fuchs, Flor & Bekrater-Bodmann 2018:1; Koleva, Ioshinov & Yoshinov 2017:3; Münger et al. 2020:581; Paul 2018:11). While a cure for this phenomenon is yet to be discovered, several strategies offer relief, namely range of motion exercises targeting the residual limb muscles in conjunction with residual limb massages, central nervous system stimulation (García-Pallero et al. 2022:63), transcutaneous nerve stimulation, acupuncture, biofeedback (Kaur & Guan 2018:367–368; Stockburger et al. 2016:162) and virtual reality therapy (Herrador Colmenero et al. 2018:297).

### Theme 2: Psychological issues

This theme highlights the psychological trauma endured post-amputation.

Amputation has significant effects on mental health. Limb loss is tantamount to the loss of a loved one and so is the grief process, as feelings of disbelief, shock and anger set in (Össur 2017:7). This was apparent in the response of participant AP3 who reported that a part of her body was lost:

‘I felt like a part of my body was lost. I was sad and fearful because I was worried about how I was going to do things for myself … Speaking to my friends and family really helped me to overcome those negative feelings of myself.’ (AP3, Female, 62)

These findings mirror those in a study conducted in Nepal (Järnhammer et al. 2017:1430). Grieving is an essential component of coping with adjustment to the amputation (Varga & Gallagher 2020:187). Furthermore, amputation is associated with the loss of independence, which stirs up feelings of depression, anger and frustration. Such emotions are evident from the responses of several participants, namely AP1 and AP12, who felt like a burden to their families, which generated feelings of guilt, despair, worry and despondency in trying to cope with relying on their family members:

‘I felt low to rely on someone else because I have never needed to rely on someone before. It was difficult for me even though my family was very supportive.’ (AP1, Male, 70)

‘I was very depressed … I just wanted to be alone, and I didn’t want anyone to see me because they will judge me and look at my amputation.’ (AP12, Male, 25)

In Singapore, participants of a qualitative study stated that the amputation restricted their physical ability to fulfil their familial roles as spouse, parent and/or sole provider, which resulted in feelings of futility and incompetence (Zhu et al. 2020:3). Similar psychological distress was evident in studies conducted in Ghana (Amoah et al. 2018:2) and Gauteng (Godlwana & Stewart 2013:49).

Considering individuals with limb loss no longer look the same as able-bodied individuals, the amputation can potentially disturb a person’s body image. If the amputee’s perception of themselves changes, it will influence how they believe they will be viewed by the public. As a result, self-esteem levels and participation in society are affected. According to Montesinos-Magraner et al. (2016:5), an altered body image and social restrictions are associated with depression and anxiety. One participant reported isolating from society in fear of how he may be perceived. Most participants expressed feelings of reduced self-esteem and confidence levels and vulnerability through their responses. Uncertainty of the future overwhelmed the participants as they communicated their doubts of the scope of their physical abilities and functional recovery. These findings were confirmed by those in studies performed in Romania (Roșca et al. 2021:4) and Singapore (Zhu et al. 2020:4).

Seeking professional guidance to cope with psychological distress helped participant AP10 to successfully adjust to the amputation. Participant AP1 described how being resilient and having a positive mindset facilitated acceptance of the amputation:

‘I did not look at myself the same and was doubtful of whether I’d walk again … I used to go for counselling. It helped me to accept the amputation.’ (AP10, Male, 53)

‘I was never embarrassed. Being strong-willed helped me a lot.’ (AP1, Male, 70)

Self-motivating and envisioning a positive future after rehabilitation to return to pre-amputation hobbies and activities can help amputees to regain a sense of normality (Roșca et al. 2021:8).

Participant AP14 expressed gratitude for the amputation as it relieved the pain in that extremity:

‘I was never self-conscious of my amputation because I was in a lot of pain. I did not have time to worry about how others were looking at me. It did not bother me either.’ (AP14, Male, 71)

Literature suggests that this response is not surprising as several individuals favour the amputation compared to the pre-operative wound, which is accompanied by debilitating pain (Amoah et al. 2018:2; Godlwana & Stewart 2013:50).

### Theme 3: Adaptation to daily activities

As evident in this theme, adaptations and adjustments had to be made in order for amputees to execute their daily tasks. Modifications included the addition of a shower stool in order to reduce the risk of falling in the shower. Altering the way in which one performs routine tasks can engender feelings of uncertainty, fear and uneasiness:

‘I have to shower while sitting on a chair … It is just different … I fell a lot in the bathroom and around the house, but I learnt to work slower and forget everything I knew before. You have to start fresh and adjust or adapt. If you get distracted, you may fall. I have learnt to even use my forehead against the wall to balance.’ (AP1, Male, 70)

Jayakaran et al. (2019:121) confirmed these findings with participants in New Zealand, who had handrails and anti-slip floors installed, and utilised shower stools in the shower as safety precautions to decrease the risk of falling and slipping. Executing daily tasks had to be relearnt and required the amputees to be vigilant. Lack of attentiveness could precipitate a fall (Godlwana & Stewart 2013:50). Participant AP1 described how he uses his head to balance on the wall to provide stability and improve balance. Similarly, participants of a study performed in Gauteng, indicated that modifying the way in which they performed tasks mitigated their frustrations and the risk of falling (Godlwana & Stewart 2013:50).

Participants AP6 and AP5 communicated the use of assistive devices to mobilise on uneven terrains in their yards, transfer to and off the toilet seat and into the bath and/or shower:

‘I use the walker to go to the bathroom and toilet, to transfer to the chair and then onto the toilet. It is not difficult.’ (AP6, Male, 70)

‘If I need to get into the car, I go up the driveway in the wheelchair first.’ (AP5, Male, 56)

Assistive devices and mobility aids, namely crutches, wheelchairs and walkers, are commonly used to enhance the performance of daily tasks, reduce injuries and navigate their environment (De-Rosende Celeiro, Sanjuán & Santos-del-Riego 2017:1803).

Ambulating on stairs is a challenging manoeuvre for amputees as it could precipitate a fall. For this reason, participant AP7 indicated that she is no longer able to fulfil her household responsibility of fetching water from the river because of the encumbrance of stairs:

‘I used to fetch water from the river but now there are too many stairs so I cannot.’ (AP7, Female, 74)

For individuals residing in rural areas, fetching water from the river is required for consumptions, domestic chores and personal hygiene. With an amputation, individuals would have to rely on other family members to takeover this responsibility (Manig 2018:57). Similarly, in Romania, it was found that the lack of functional independence to perform daily activities and resume previous responsibilities engendered feelings of frustration, bitterness and anger (Roșca et al. 2021:8). De-Rosende Celeiro et al. (2017:1804) suggested that rehabilitation programmes aimed at assisting amputees to perform activities of daily living post-operatively should be established as it will foster self-sufficiency and improve safety.

### Theme 4: Familial obligations and responsibilities

One of the themes derived from the findings was familial obligations and responsibilities. The participants described the impact of receiving support from their family members and friends:

‘My family would always tell me that I am not alone, and I am only human.’ (AP8, Male, 65)

‘I did feel like a burden in the beginning because they were doing almost everything for me. My wife and my daughter are my two crutches.’ (AP1, Male, 70)

Some amputees may feel lonely post-amputation as their family members will not fully understand the gravity of the physical and emotional obstacles associated with amputation (Paul 2018:10). As evidenced in the data, most participants received immense emotional and physical support from their families. Participant AP1 described his wife and daughter as his ‘two crutches’, implying that they were his source of physical support when he endured physical challenges post-operatively. However, the lack of mobility of the amputees placed the load of additional responsibilities on family members, which left participant AP1 feeling like a burden. This is a common feeling reported in several studies (Amoah et al. 2018:3; Järnhammer et al. 2017:1432; Roșca et al. 2021:8).

The literature is replete with evidence of supportive family members and friends (Godlwana & Stewart 2013:51; Stutts et al. 2015:749). A study in the United States (US) found that pets were valuable sources of support in helping the amputee participants to cope with the amputation (Stutts et al. 2015:749). Individuals with limb loss express great appreciation towards friends and family for the support received. In Gauteng, the amputation was found to improve relationships between spouses or partners and/or their family members (Godlwana & Stewart 2013:51).

Participant AP4 stated that he acknowledged his family’s emotions and helped them to understand and accept his amputation:

‘They were ashamed in the beginning, but I spoke to them about it.’ (AP4, Male, 40)

Manig (2018:79) emphasised that family counselling is crucial to educate and equip the family with coping strategies for the new household dynamics.

In contrast, the amputation may place strain on some relationships especially when the amputees feel misunderstood or uncared-for by their spouses (Stutts et al. 2015:749). Some participants did not receive the support they expected from their family members which generated feelings of disappointment and sorrow. Participant AP13 decided to prioritise her well-being and personal growth after feeling neglected by her family:

‘My family was quite supportive initially … I do feel lonely at times and that my family just abandoned me. But I have decided to put myself first.’ (AP13, Female, 65)

Similarly, in Singapore, participants felt displeased and angry towards their unsupportive family members (Zhu et al. 2020:4). When amputees do not receive the familial support they initially expect, feelings of insecurity can arise, which requires that they receive professional assistance (Uytman 2014:13).

### Theme 5: Stereotyping in society

The final theme that emerged from the findings was stereotyping in society. The participants described the negative comments from individuals of the public:

‘Sometimes people in public will call me names such as hop-a-long or hop-a-long Cassidy.’ (AP2, Male, 58)

Participants reported on the stigma attached to amputation from the encounters with some members of the public. Participant AP2 was insulted and had demeaning words directed at him. Despite the participant being unperturbed by the negative remarks, a clear indication of the stigma and discrimination that exists towards those with physical disability is evident.

Participant AP12 was driven into self-isolation as he felt embarrassed, inferior and self-conscious:

‘It made me feel like nobody will love me for who I am. Initially their opinions affected me to the point where I never left my house but after some time you adjust and accept the situation.’ (AP12, Male, 25)

However, with time participant AP12 accepted his amputation and was able to positively re-integrate into society. The social stigma attached to amputation were also highlighted by amputee participants in Singapore (Zhu et al. 2020:4), Spain (Montesinos-Magraner et al. 2016:5) and South Africa (Godlwana & Stewart 2013:50). According to Montesinos-Magraner et al. (2016:5), isolating from public areas also reduces the chances of amputees starting and establishing romantic relationships.

Participant AP5 initially felt insecure and used clothing that masked his amputation:

‘People staring used to make me feel very self-conscious and aware. I started to only use long pants and jeans so that nobody would notice anything different … I stopped caring about what people thought … My mind was focussed on getting better and getting the prosthesis.’ (AP5, Male, 56)

Shifting his focus from the constant stares of the public to obtaining his prosthesis instilled hope and enthusiasm. This echoed a response in another study, where the participant was unaffected by society’s opinion and rather more interested in receiving the prosthesis (Zhu et al. 2020:4).

While there are some facilities in public transport vehicles for individuals with disability, members of the public become antagonised and prevent amputees from using them. Participant AP7 described how a member of the public in the taxi refused to give up the passenger seat for her:

‘Amputations are quite popular so many people do not really take notice or get shocked … I do not have to wait in lines and that causes problems because the other people get angry. I get to sit in the front of the taxi or behind the driver and if someone is sitting there, they do not want to move.’ (AP7, Female, 74)

These facilities ensure the amputees safety and facilitate mobility. It reflects the insensitive and hostile nature of some individuals in society who lack understanding of the needs of amputees. Participants in a study conducted by Stutts et al. (2015:747) in the US also reported experiences of public discrimination. Contrary to this study’s findings, the participants of Godlwana and Stewart’s (2013:50) study reported receiving sympathy from members of the public.

A significant finding that emerged from the data was the unmatched support received from other amputees:

‘It also helped to have friends that were amputees, we were all going through the same things.’ (AP9, Female, 45)

Evidence indicates that amputees become more content with life after receiving support from other individuals with limb loss or attending amputee support groups (Williams 2016:104).

Participant AP14 indicated that he offers encouragement to and coping strategies with other amputees by sharing his experiences of adapting to and accepting the amputation:

‘I was a speaker at this one place for other amputees. I would encourage them and speak about my experiences. People treating me normally encouraged me to accept my amputation and be confident.’ (AP14, Male, 71)

Several studies reflect the importance of amputee support groups, as the participants report having positive experiences that are quintessential facets of their adjustment process (Stutts et al. 2015:750). The support groups mitigate loneliness and alleviate anxiety, depression and stress that subsequently enhances mental health (Abu Shawish et al. 2021:531).

### Limitations

While this study provided significant data regarding the quality of life of individuals with limb loss, it was not without limitations.

The current study’s amputee participants resided in the urban regions of KwaZulu-Natal. Further research should explore the biopsychosocial challenges experienced by amputees in rural regions. Such data will provide insight into the specific rehabilitation services and care required and will enable the provision of these services in outlying areas to be operational.

### Recommendations

All orthopaedic surgeons should have a system in place in which the individual undergoing amputation and their family members receive resources, such as brochures or websites, providing information on living with amputation and interdisciplinary services available to them. Moreover, psychologists and social workers should be incorporated into the care plan after surgery to provide psycho-therapeutic support. Receiving psychological help will offer an avenue for the amputee to cope with mental distress while receiving professional guidance on how to successfully adjust to and accept the amputation. As evidenced by Williams (2018:104), individuals with limb loss experience greater satisfaction with life after receiving one on one and group social support. Therefore, physical disability support groups should be established in medical facilities throughout the country to promote psychosocial well-being.

Future research should focus on various coping strategies and new techniques for relief of phantom limb pain, vocational reintegration and how places of work can accommodate individuals with disability, enhancing rehabilitation programmes to provide holistic forms of care, improving transportation services to healthcare facilities for individuals with limb loss at no cost and establishing programmes to educate individuals with limb loss and their family members on the amputation experience inclusive of management strategies and appropriate healthcare services.

## Conclusion

This study explored the biopsychosocial effects of a transtibial amputation, which are illuminated through the diverse range of challenges amputees experienced post-amputation. Phantom limb pain, limited functional mobility and adapting to perform daily tasks were among the main physical effects reported by the amputee participants. The findings indicate that losing a limb gives birth to several psychological issues, namely grief, a lack of independence, reduced self-esteem, body image anxiety, depression, self-isolation and fear of the future. The findings corroborate with those internationally and add to the limited body of knowledge in South Africa related to amputation. The importance of psychological intervention and a strong support network was brought to the fore. While familial support facilitated the adjustment process, the amputees indicated that receiving support from other amputees would be unmatched in mitigating the various psychological effects.

Future research could bring in data on the experiences of the amputee’s support network and establish psychosocial programmes to educate, counsel and comfort the amputee and their carers. Regardless of the challenges experienced, being resilient and having a positive mindset was reported to promote successful adjustment to the amputation. Having thoroughly explored the biopsychosocial effects of amputation, a holistic wellness programme incorporating various tools can prove beneficial for future individuals with limb loss to receive as a mandatory care procedure. Policy needs to be revised to ensure that post-surgery, medical facilities provide physical support to the individual and psychosocial support to them and their family members.

The primary intention of the United Nations Convention on the Rights of Persons with Disabilities (United Nations General Assembly 2007) is to protect, ensure and promote the full and equal enjoyment of all human rights and freedoms by individuals with disability and further to uphold their autonomy and dignity. This study recognised the stigma attached to amputation as some participants described that society often engaged discrimination, unwarranted hostility and excluding them. It is imperative that the general public become aware of amputation and its effects on amputees so as to ensure their reintegration into society. Moreover, there should be educational campaigns hosted throughout the country specifically in rural areas, representation of individuals with disability in media in various settings outside of a disability context, promotion of receiving psychosocial support and media coverage of positive and fulfilling testimonies attached to life with an amputation.
